# An Interview-Based Assessment of the Experience of Cognitive Impairment in Multiple Sclerosis: The Cognitive Assessment Interview (CAI)

**DOI:** 10.3389/fneur.2021.637895

**Published:** 2021-02-11

**Authors:** Tehila Eilam-Stock, Pamela Best, Kathleen Sherman, Michael T. Shaw, Joseph Ventura, Lauren B. Krupp, Leigh E. Charvet

**Affiliations:** ^1^Department of Neurology, New York University Grossman School of Medicine, New York, NY, United States; ^2^Department of Psychology, Binghamton University, Binghamton, NY, United States; ^3^Department of Psychiatry and Biobehavioral Sciences, Semel Institute for Neuroscience and Human Behavior, University of California, Los Angeles, Los Angeles, CA, United States

**Keywords:** multiple sclerosis, cognitive, neuropsychological, cognitive assessment interview, CAI, daily functioning

## Abstract

**Background:** Cognitive impairment is a common feature of multiple sclerosis (MS). A semi-structured interview, including informant input, can characterize the experience of individuals living with MS and cognitive involvement.

**Objective:** We administered the Cognitive Assessment Interview (CAI), a patient- and informant-based semi-structured interview, to characterize the experience of cognitive impairments in those living with MS.

**Methods:** Trained raters administered the CAI to a sample of MS participants and their informants enrolled for a trial of cognitive remediation. Cognitive impairments on the CAI were characterized and compared to those captured by neuropsychological and self-report measures.

**Results:** A total of *n* = 109 MS participants (mean age = 50.3 ± 12.2) and their available informants (*n* = 71) were interviewed. Participants reported experiencing processing speed (90/106, 85%), working memory (87/109, 80%), and learning and memory (79/109, 72%) problems most commonly. CAI-based ratings were moderately correlated with a self-report measure (Multiple Sclerosis Neuropsychological Screening Questionnaire, *r*_s_ = 0.52, *p* < 0.001) and only mildly correlated with objective neuropsychological measures specific to executive functions (*r*_*s*_ = 0.21, *p* = 0.029). For those with informant interviews, ratings were overall consistent, suggesting that the CAI is valid even in cases in which an informant is unavailable and the interview is conducted with the patient alone (as is often the case in clinical and research settings).

**Conclusions:** The CAI provides a semi-structured interview to characterize the experience of cognitive impairment in MS, with findings representing real-world functioning, adding valuable information to both self-report measures and neuropsychological assessment.

## Introduction

Cognitive difficulties affect up to 70% of individuals living with multiple sclerosis (MS) (1, 2) and are associated with significant disability and overall reduction in quality of life (3, 4). Objective impairments are most commonly found in the domains of processing speed and efficiency, complex attention and working memory, and novel learning (1, 2, 5). A semi-structured interview, in which a patient's self-report is reviewed by the expert judgment of a skilled interviewer and corroborated with a report of a significant other who lives with the patient and observes them daily, can be an important measure for fully characterizing the impact of cognitive impairments on daily cognitive functioning.

Structured and semi-structured interviews are considered the gold standard accompaniment to objective cognitive measures in other disorders with cognitive involvement, and specifically for use in understanding the experience of age-related dementias, including the Clinical Dementia Rating (CDR) scale (6, 7), the Interview for Deterioration in Daily Living Activities in Dementia [IDDD; (8)], and the Disability Assessment for Dementia [DAD; (9)]. Items in these scales probe the patient's ability to complete activities of daily living, such as bathing, dressing, eating, attending community events, and using the telephone. However, the items in these scales are designed for those of older age and with more advanced forms of cognitive impairment, therefore resulting in a “ceiling effect” for many with MS, often missing the impact of milder and more subtle areas of impairment.

The Cognitive Assessment Interview [CAI; (10)] was developed to assess daily functioning in patients with schizophrenia, who in comparison to those with age-related dementias are typically of a younger age and have a higher level of cognitive functioning, with domains of involvement including working memory, attention, verbal learning and memory, reasoning and problem solving, speed of processing, and social cognition, that may be more applicable for those with MS. The CAI consists of 10 semi-structured interview questions and is conducted by a trained clinical rater with a patient, as well as with an informant (e.g., caregiver or spouse) when available. The interview ratings (provided by the clinical rater) are based on the patient's and informant's responses and examples. The CAI has demonstrated good test–retest and interrater reliability, high internal consistency, and significant correlation with functional and objective cognitive measures (10–13).

Several self-report measures of daily cognitive functioning were developed for use in MS, including the Multiple Sclerosis Neuropsychological Screening Questionnaire [MSNQ; (14, 15)] and the perceived deficits questionnaire [PDQ; (16)]. These questionnaires provide useful insight into a patient's perception of their cognitive difficulties. However, they may not accurately reflect the true level of cognitive impact on daily functioning, due to either over- or underestimation of subjective experiences and the influence of mood states in ratings [e.g., depression; (14–17)]. The CAI has been shown to be independent from depressive symptoms (11), suggesting that raters were effectively able to differentiate their participants' cognitive complaints from mood symptoms.

The aim of the current study was to utilize the CAI to characterize the experience of cognitive impairment for people living with MS. Given its rating of relevant cognitive domains (e.g., processing speed and working memory), suitability for use in younger and higher functioning patients, and utilization of informant input, we hypothesize that the CAI is suitable for additional characterization of impairment, separate from objective neuropsychological and self-report measures. To test our hypothesis, we administered the CAI to a large sample of people with MS reporting cognitive difficulties, as well as to their informants where possible, and characterized daily cognitive impairment in MS based on its findings. We additionally compared CAI findings to performance on a battery of neuropsychological measures sensitive to MS-related cognitive impairment, as well as an objective measure [the Test of Everyday Cognitive Ability (TECA); (18)] and a self-report measure of daily cognitive functioning (i.e., MSNQ).

## Methods

### Participants

Participants were enrolled in a clinical trial of a cognitive remediation program (19). All participants had a confirmed diagnosis of MS [all subtypes were included; (20)] and had at least mild cognitive impairment as defined by an age-normative *z* score of −1.0 or lower on the symbol digit modalities test (SDMT). Participants were also required to have an estimated premorbid functioning in the normal range, based on a standard score of 80 or above on a reading recognition test [the Wide Range Achievement Test, third edition; WRAT-3; (21)] as a proxy for premorbid intellectual functioning (22). Exclusion criteria included a history of developmental disorders, conditions other than MS that may cause cognitive impairment, a primary psychiatric disorder, a substance use disorder, any other major medical disorder, and relapse or steroid use in the month prior to enrollment.

All participants provided written informed consent to study procedures that were approved by the Institutional Review Board and the Committee on Research Involving Human Subjects at Stony Brook Medicine, Stony Brook, New York, and in compliance with the Helsinki Declaration.

### Cognitive Assessment Interview

Two clinical raters completed standardized training for CAI administration (provided by Dr. Ventura) and met consensus for rating of example recorded video interviews. The CAI consists of 10 items addressing six cognitive domains (Table 1): Attention/Concentration, Working Memory, Verbal Learning and Memory, Reasoning and Problem Solving, Speed of Processing, and Social Cognition. Raters interviewed participants and available informants separately. Informants were defined as someone identified by the participant who lives with them and is familiar enough to comment on their cognitive and daily functioning (spouse, partner, caregiver, or adult child).

**Table 1:** Cognitive Assessment Interview (CAI) summary.

**CAI item**	**Cognitive domain**
**Item 1:** Difficulty maintaining newly learned information in mind for brief periods (long enough to use)?	Working memory
**Item 2:** Difficulty performing “on the spot” mental manipulations or computations?	
**Item 3:** Problems sustaining concentration over time (without distraction)?	Attention/Concentration
**Item 4:** Difficulty focusing on select information (if there is not obvious distraction)?	
**Item 5:** Trouble learning and remembering verbal material?	Verbal learning and memory
**Item 6:** Difficulty recalling recent events?	
**Item 7:** Lack of flexibility in generating alternate plans when needed?	Reasoning and problem solving
**Item 8:** Problems in situations requiring judgment?	
**Item 9:** Performs tasks slowly?	Speed of processing
**Item 10:** Difficulty appreciating another person's intentions/point of view?	Social cognition
**Global severity score**	Global cognitive functioning
**Global assessment of functioning score**	

Each domain is rated according to the presence and severity of impairment based on the participant and informant (when available) responses to semi-structured questions and prompts for examples of cognitive difficulties from 1 (no impairment) to 7 (severe impairment). In addition, the clinical rater provides an overall global severity (GS) score of cognitive impairment, from 1 to 7, and global assessment of functioning cognition ratings (GAF-Cog) ranging from 0 to 100 (with 0 being most severely impaired and 100 being the most highly functional).

### Cognitive Measures

Serving as the baseline evaluation for the clinical trial, participants completed an objective battery of tests addressing cognitive domains similar to those addressed with the CAI, including working memory, attention, learning and memory, executive functions, and processing speed (see Table 2 for summary of tests and domains). Briefly, the Digit Span Backward condition (Digit Span subtest) and the Letter–Number Sequencing subtest from the Wechsler Adult Intelligence Scale, 4th Edition [WAIS-IV; (23)] were used as measures of working memory. The WAIS-IV Digit Span Forward condition from the Digit Span subtest was used as a measure of attention. Verbal learning was assessed with the learning trials on the Selective Reminding Test [SRT; (25)], and visual learning was assessed with the learning trials on the Brief Visuospatial Memory Test—Revised [BVMT-R; (26)]. The Trail Making Test, Alternating Numbers and Letters condition from the Delis-Kaplan Executive Function System [D-KEFS; (27)] was used to assess executive functions. Finally, the Paced Auditory Serial Addition Test [PASAT; (28)], 2 seconds condition, and the SDMT measured information processing speed. While no objective test of social cognition was administered, measures of complex information processing speed (i.e., PASAT and SDMT) were also used to compare to the social cognition domain on the CAI, based on previous studies demonstrating a strong link between these cognitive functions (30).

**Table 2:** Summary of neuropsychological measures and Cognitive Assessment Interview (CAI) items for each of the cognitive domains on the CAI.

**Cognitive domain**	**Neuropsychological measures**	**CAI items**
Working Memory	Wechsler Adult Intelligence Scale, 4th Edition (WAIS-IV)^a^: Digit Span Backward and Letter–Number Sequencing subtests	1, 2
Attention	WAIS-IV^a^: Digit Span Forward	3, 4
Learning and Memory	Selective Reminding Test (SRT)^b^	5, 6
	Brief Visuospatial Memory Test—Revised (BVMT-R)^c^	
Executive Functioning	Delis-Kaplan Executive Function System (D-KEFS)^d^: Trail Making Test, Alternating Numbers and Letters Condition	7, 8
Processing Speed	Paced Auditory Serial Addition Test (PASAT): 2 s condition^e^	9
Social Cognition	Symbol Digit Modalities Test (SDMT)^f^	10

a *Wechsler (23); Drozdick et al. (24)*;

b *Buschke (25)*;

c *Benedict and Groninger (26)*;

d *Delis et al. (27)*;

e *Diehr et al. (28)*;

f *Smith (29)*.

For all cognitive measures, raw scores of each participant were converted into age-normed *z* scores. In cases where two measures were used to assess one cognitive domain (i.e., working memory, learning and memory, and processing speed), *z* scores from both tests were averaged into one domain score. Normative *z* scores were also averaged across all domains to obtain a composite *z* score to serve as a measure of global cognitive functioning.

As an objective measure of real-world functioning, the TECA (18) was also administered. The TECA is a 10-item test of timed instrumental activities of daily living (e.g., reading a grocery list, counting change) developed for use in MS. For a subjective measure of functioning, participants completed the MSNQ, a self-report measure of daily cognitive functioning (14).

### Statistical Analysis

We first characterized the sample based on CAI findings. Mean and standard deviations were computed for the sample's demographic and clinical characteristics. To characterize daily cognitive impairments in our sample based on CAI ratings, means, standard deviations and frequencies of global cognitive impairment, as well as impairment within the CAI domains were calculated. Finally, given that in MS, not all participants will have an available informant for interview, we also tested the consistency of ratings between the participant and informant interviews. Thus, intraclass correlation coefficient (ICC) estimates and their 95% confident intervals were calculated between participant-based and informant-based ratings to better capture the relationship between the two.

To further examine the additive value of the CAI to traditional objective and self-report measures, we compared impairment, as measured by the CAI, to that identified by the neuropsychological measures, the TECA, and the MSNQ. As the CAI is measured on ordinal scales, data do not meet the assumptions for parametric statistics. Thus, non-parametric Spearman rank correlations were calculated between (1) global CAI and neuropsychological measures (including the TECA), (2) specific cognitive domains on the CAI and neuropsychological tests, and (3) global CAI indices and the MSNQ. All statistical analyses were performed using SPSS statistical package version 25.0 (31).

## Results

A total of *n* = 109 individuals with MS were interviewed using the CAI and assessed with an objective neurocognitive test battery. The majority of CAIs also included an informant (*n* = 71). Participants ranged in age from 18 to 69 (mean = 50.3 ± 12.2) years, were 78% female, and included those with relapsing remitting (64.2%) and progressive (31.2%) subtypes. See Table 3 for full demographic and clinical characteristics of the sample.

**Table 3:** Demographic and clinical features of the sample.

**Patients (*n* = 109)**	
Mean age (range)	50.3 (18–69)
Mean years education (range)	14.8 (11–20)
Percent female	78%
**Race** ***n*** **(%)**
Caucasian	92 (84.4%)
African-American	8 (7.3%)
Unspecified	7 (6.4%)
**Diagnosis** ***n*** **(%)**
Relapsing remitting MS	70 (64.2%)
Secondary progressive MS	28 (25.7%)
Primary progressive MS	6 (5.5%)
Not reported	5 (4.6%)
Median EDSS^a^ (range)	3.5 (0–8.5)
Mean WRAT^b^ standard score (range)	103.6 (80–119)
Mean SDMT^c^ z score (SD)	−2.10 ± 0.99

a *The expanded disability status scale*;

b *The wide range achievement test*;

c *The symbol digit modalities test*.

### CAI Ratings of the Experience of Cognitive Impairment in Daily Life

#### Global Impairment

Global severity ratings indicated at least minimal impairment (defined as a score of 2 or greater) in 92% of the sample (95/103, mean rating = 2.78 ± 1.0). Global assessment of functioning was at least mildly impaired in 50% of the sample (defined as rating score ≤ 70; mean rating = 74.0 ± 14.0), including 29% with mild impairment (GAF-Cog = 61–70), 19% with moderate impairment (GAF-Cog = 51–60), and 2% with severe impairment (GAF-Cog ≤ 50).

#### Frequency and Severity of Impairment Across Domains

**Figure 1:**
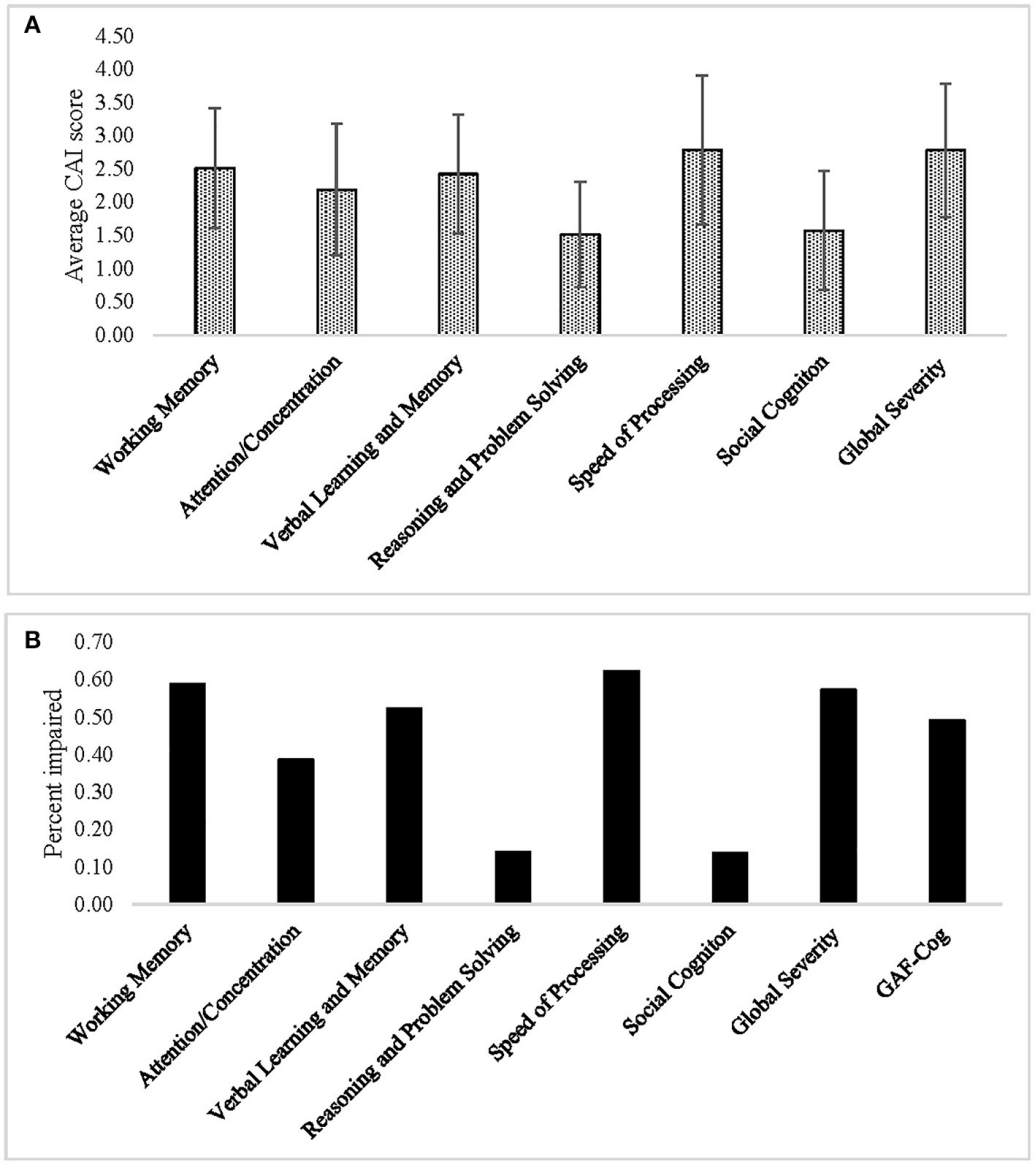
**(A)** Average ratings and **(B)** percent impairment for each of the Cognitive Assessment Interview (CAI) cognitive domains, as well as for the Global Severity and Global Assessment of Functioning (GAF-Cog) scales.

The domains with the highest percentage of impairment (scored as 2 or greater) were Speed of Processing (90/106, 85%, mean rating = 2.78 ± 1.12), followed by Working Memory (87/109, 80%, mean rating = 2.51 ± 0.9), Verbal Learning and Memory (79/109, 73%, mean rating = 2.42 ± 0.9), and Attention/Concentration (64/109, 59%, mean rating = 2.19 ± 0.99), while the domains of Reasoning and Problem Solving and Social Cognition were less affected in our sample (28 and 37%, respectively) (Figure 1, Table 4). Among those individuals with any impairment (rated >1), the overall severity level was rated as mild (mean = 2.57 ± 0.83).

**Table 4:** Frequency of impairment across the different Cognitive Assessment Interview (CAI) domains in the sample.

	**Cognitive domain**						
	**Working memory**	**Attention/Concentration**	**Verbal learning and memory**	**Reasoning and problem solving**	**Speed of processing**	**Social cognition**	**Global severity score**
No impairment (1–1.5)	20.2% (*n* = 22)	41.3% (*n* = 45)	27.5% (*n* = 30)	72% (*n* = 77)	15.1% (*n* = 16)	63.3% (*n* = 69)	7.8% (*n* = 8)
Minimal (2–2.5)	45.9% (*n* = 50)	33.0% (*n* = 36)	35.8% (*n* = 39)	23.4% (*n* = 25)	22.6% (*n* = 24)	22.9% (*n* = 25)	35.0% (*n* = 36)
Mild (3–3.5)	22.0% (*n* = 24)	14.7% (*n* = 16)	32.1% (*n* = 35)	3.7% (*n* = 4)	38.7% (*n* = 41)	8.3% (*n* = 9)	34.0% (*n* = 35)
Moderate (4–4.5)	11.0% (*n* = 12)	10.1% (*n* = 11)	3.7% (*n* = 4)	0.9% (*n* = 1)	16.0% (*n* = 17)	4.6% (*n* = 5)	18.4% (*n* = 19)
Severe (5–7)	0.9% (*n* = 1)	0.9% (*n* = 1)	0.9% (*n* = 1)	0% (*n* = 0)	7.5% (*n* = 8)	0.9% (*n* = 1)	4.9% (*n* = 5)
Total impairment	79.8% (*n* = 87)	58.7% (*n* = 64)	72.5% (*n* = 79)	28% (*n* = 30)	84.9% (*n* = 90)	36.7% (*n* = 40)	92.2% (*n* = 95)

### Consistency of Ratings Between Participant and Informant Interviews

Among those participants who also had informant interviews (*n* = 71), intraclass correlations were calculated between participant-based and informant-based ratings. ICC estimates and their 95% confident intervals were calculated based on an average-rating, absolute-agreement, 2-way random-effects model. The ICC between participant-based and informant-based ratings was fair [ICC = 0.67, 95% CI = 0.62 to 0.72, *F*_(628, 628)_ = 3.10, *p* < 0.001]. On average, participant-based ratings indicated higher severity on daily tasks requiring working memory (mean rating = 3.14 ± 1.17), verbal learning and memory (mean rating = 2.58 ± 1.25), and information processing speed (mean rating = 2.74 ±1.28), while informant-based ratings were in the minimal range of severity in these domains. Interestingly, informant-based ratings indicated, on average, some difficulty in cognitive flexibility (mean rating = 1.59 ± 0.97) and social cognition (mean rating = 1.65 ± 1.14), while participant ratings indicated, on average, no impairment in these domains.

### Correspondence Between CAI and Objective Neuropsychological Assessment Measures

To test correspondence between the CAI and objective neuropsychological measures on global cognitive functioning, Spearman's rank-order correlation analysis was performed between the CAI global indices (GS and GAF-Cog) and the neuropsychological global measures (composite *z* score and TECA). While TECA and composite *z* scores significantly correlated with each other (*r*_s_ = −0.58, *p* < 0.001), no significant correlations were identified between the GS or GAF-Cog and composite *z* scores (GS *r*_s_ = −0.13, *p* = 0.195; GAF-Cog *r*_s_ = 0.17, *p* = 0.082). Similarly, there were no significant correlations between GS or GAF-Cog and the TECA (GS *r*_s_ = 0.05, *p* = 0.646; GAF-Cog *r*_s_ = −0.13, *p* = 0.186).

To test correspondence between the CAI cognitive domains and objective neuropsychological domains, a Spearman's rank-order correlation analysis was performed between ratings of CAI individual cognitive domains and the participants' performance on the corresponding objective neuropsychological domains (Table 2). Results indicated a mild correlation between CAI ratings and the participant's cognitive performance in the domain of executive functions (e.g., reasoning and problem solving; *r*_s_ = −0.21, *p* = 0.033). Executive function performance was also mildly correlated with the GAF-Cog (*r*_s_ = −0.21, *p* = 0.029). No other statistically significant correlations were identified between the CAI and neuropsychological measures for any of the other domains.

### Correspondence Between CAI and MSNQ

Spearman's rank-order correlation analysis was performed between the CAI global scores (GS and GAF-Cog scales) and the MSNQ, a self-report questionnaire assessing daily cognitive functioning (*n* = 103). Moderate correlations were identified between the MSNQ and patient ratings on both global indices of the CAI (GS *r*_s_ = 0.52, *p* < 0.001; GAF-Cog *r*_s_ = −0.43, *p* < 0.001). The MSNQ did not significantly correlate with either the composite cognitive *z* scores (*r*_s_ = −0.09, *p* = 0.365) or the TECA scores (*r*_s_ =0.08, *p* = 0.448).

### Discussion

This is the first study to use a semi-structured interview and include informant input to characterize the experience of cognitive impairment in a large sample of individuals with MS. In this MS sample of individuals meeting objective (SDMT) criteria for at least mild cognitive impairment, the CAI also indicated an overall mild cognitive impairment. Consistent with the expected areas of cognitive difficulties in MS (1, 2, 5), ratings on the CAI indicated that the most frequent experience of cognitive impairments were in the areas of processing speed, working memory, and verbal learning and memory (affecting more than 70% of the sample). Processing speed, specifically, was the leading area of difficulty, affecting 85% of our sample. It has been argued that impaired processing speed is among the earliest cognitive functions to be affected by MS (32) and is thought to be related to deterioration of white matter integrity, affecting signal transmission speed and efficiency within and between brain networks (33, 34). In addition, it has been proposed that slowed information processing speed underlies other MS-related cognitive impairments, including working memory and novel learning (35, 36). Our findings expand the existing literature, demonstrating that slowed processing speed is a main area of difficulty affecting daily functioning in a large majority of individuals with MS with cognitive involvement.

As not all MS participants have an available informant for interview (e.g., those who live alone), we evaluated the contribution of the informant interview in the ratings of the MS patients. For the sample subset with informant interviews, ratings were overall consistent, suggesting that the CAI is valid even in cases in which informant is unavailable and interview is conducted with the patient alone (as is often the case in clinical and research settings). However, participant-based ratings indicated elevated levels of impairment on items assessing working memory, learning and memory, and information processing speed, compared to informant-based ratings. These findings correspond to the description of cognitive involvement in MS as an “invisible” or “hidden” symptom of the disease (37, 38), with patients often expressing that even the people who are closest to them (i.e., caregivers) underestimate the extent to which cognitive difficulties can affect their everyday functioning and quality of life. Conversely, informant-based ratings indicated some difficulty in reasoning and problem solving and in social cognition, while participant-based ratings indicated no impairment in these domains, suggesting that these are more “visible” cognitive manifestations that are more readily apparent to the patient's environment. Indeed, unlike working memory, learning and memory, and processing speed, these functions involve others to a greater extent. It is possible that the reduced self-awareness of patients to these cognitive changes may stem from an attribution error, as these difficulties are easier to attribute to the external environment, rather than to the self.

Across neuropsychological testing domains, executive functioning performances had the strongest correspondence to CAI findings and therefore may be most predictive of the experience of day-to-day cognitive functioning. In addition, CAI findings were moderately correlated with the subjective self-report measure, indicating that the interview-based format can provide additional and fuller detail than captured by a self-reported rating. Together, our findings suggest that the CAI can provide a unique characterization of the patient's experience of cognitive difficulties that may be distinct from what is captured by objective neuropsychological assessments and self-report measures. While neuropsychological tests are “clean” measures of specific cognitive domains administered in well-controlled settings, and self-reports offer an entirely subjective experience of cognitive difficulties by the patient, the CAI uniquely offers a more objective assessment of the patient's daily cognitive difficulties. Our finding that the TECA was significantly correlated with the composite score of objective cognitive measures but not with the CAI global indices or the MSNQ exemplifies this idea by demonstrating that an objective measure of daily cognitive abilities in the quiet, controlled environment of the lab or clinic is more closely related to other objectively assessed cognitive abilities rather than the patient's individual experience of day-to-day cognitive functioning in the real-world environment.

While the CAI has demonstrated good test–retest reliability and high internal consistency when administered to a sample of individuals with schizophrenia, these psychometric properties were not measured in the current study and would be important to assess in future studies. Indeed, the current work aimed to characterize, rather than validate, the CAI in MS. Nevertheless, we believe that these core psychometric qualities of the CAI would not be inherently different in our MS sample due to important characteristics shared by the two samples, such as a wide age range (including young adults), relatively subtle changes in cognitive functioning (e.g., as compared to neurodegenerative disorders), and similar cognitive domains affected by the two conditions (e.g., processing speed).

As depression and fatigue are common in MS and may affect daily cognitive functioning, one limitation of the current study is the lack of mood and fatigue measures. In addition, while the CAI has been shown to be independent from depressive symptoms in individuals with schizophrenia (11), it would be important to determine whether this finding extends to the MS population as well. Therefore, it would be essential to include these measures in future studies using the CAI to improve our understanding of the relationship between mood and fatigue and daily cognitive functioning in MS, as measured by the CAI.

## Conclusion

The present study is the first to characterize the impact of cognitive impairments on daily living in MS based on detailed interviews with a large sample of patients and caregivers. MS participants with at least mild objective cognitive impairment have overall mild CAI cognitive impairment as well, with aspects of processing speed and working memory being the most widely affected. The CAI captures aspects of real-world functioning that are distinct from both a self-reported inventory and objective cognitive testing, thus enriching the global understanding of the impact cognitive impairment may have on daily living in MS.

## Data Availability Statement

The raw data supporting the conclusions of this article will be made available by the authors, without undue reservation.

## Ethics Statement

The studies involving human participants were reviewed and approved by the Institutional Review Board and the Committee on Research Involving Human Subjects at Stony Brook Medicine, Stony Brook, New York. The patients/participants provided their written informed consent to participate in this study.

## Author Contributions

LC, LK, TE-S, PB, JV, KS, and MS wrote, reviewed, and edited the manuscript. LC, TE-S, PB, and MS wrote the original draft. TE-S and PB done visualization. LC done validation, supervision, and funding acquisition. LC, MS, and KS were responsible for resources, investigation, data curation, and project administration. LC and KS designed the methodology. TE-S, LC, and MS done the formal analysis. LC, LK, KS, and MS conceptualized the study. All authors contributed to the article and approved the submitted version.

## Conflict of Interest

The authors declare that the research was conducted in the absence of any commercial or financial relationships that could be construed as a potential conflict of interest.
